# miR-130a and miR-27b Enhance Osteogenesis in Human Bone Marrow Mesenchymal Stem Cells via Specific Down-Regulation of Peroxisome Proliferator-Activated Receptor γ

**DOI:** 10.3389/fgene.2018.00543

**Published:** 2018-11-14

**Authors:** Kanokwan Seenprachawong, Tulyapruek Tawornsawutruk, Chanin Nantasenamat, Pornlada Nuchnoi, Suradej Hongeng, Aungkura Supokawej

**Affiliations:** ^1^Department of Clinical Microscopy, Faculty of Medical Technology, Mahidol University, Nakhon Pathom, Thailand; ^2^Department of Orthopedics, Faculty of Medicine, Ramathibodi Hospital, Mahidol University, Bangkok, Thailand; ^3^Center of Data Mining and Biomedical Informatics, Faculty of Medical Technology, Mahidol University, Bangkok, Thailand; ^4^Center for Research and Innovation, Faculty of Medical Technology, Mahidol University, Nakhon Pathom, Thailand; ^5^Department of Pediatrics, Faculty of Medicine, Ramathibodi Hospital, Mahidol University, Bangkok, Thailand

**Keywords:** mesenchymal stem cell, fate determination, PPAR, miRNA, osteogenesis, osteoblast

## Abstract

Mesenchymal stem cell (MSC) is a type of stem cell that is capable of differentiating into osteoblasts and adipocytes. The pathological perturbation of MSC fate determination is well demonstrated by the replacement of bone tissues with fat in those with osteoporosis and osteopenia. Cell fate determination can be regulated by epigenetic and post-transcriptional mechanisms. MicroRNAs (miRNAs) are small endogenous non-coding RNA molecules that mediates the post-transcriptional regulation of genes expression. We hypothesized that miRNA specified to *PPAR*γ, a major transcription factor of adipogenesis, is responsible for the differentiation of MSCs into osteoblasts. Candidate miRNA that is responsible for target gene inhibition was identified from the miRNA database via bioinformatic analyses. In this study, miR-130a and miR-27b were selected for investigation on their role in specifically binding to peroxisome proliferator-activated receptor γ (*PPARγ)* via *in vitro* osteogenesis of human MSCs. During osteogenic differentiation of human MSCs, the expression level of miR-130a and miR-27b were found to be upregulated. In the meanwhile, adipogenic marker genes (*PPAR*γ and *C/EBP*β) were found to decrease, which is in contrary to the increased expression of osteogenic marker genes (*RUNX2* and *Osterix*). MSCs were transfected with mimics and inhibitors of miR-130a and miR-27b during *in vitro* osteogenesis followed by evaluation for the presence of osteogenic markers via quantitative gene expression, Western blot analysis and alkaline phosphatase activity assay. The overexpression of miR-130a and miR-27b is shown to enhance osteogenesis by increasing the gene expression of *RUNX2* and *Osterix*, the protein expression of RUNX2, COL1A1, and Osterix as well as the alkaline phosphatase activity. Taken altogether, these results suggested that miR-130a and miR-27b could promote osteogenesis in human MSCs by targeting the *PPARγ.*

## Introduction

Mesenchymal stem cells (MSCs) were first discovered by Friedenstein et al. (1966). MSCs are adult multipotent stem cells that restrain multi-cell type differentiation property, particularly, in mesodermal lineages such as osteoblasts, chondrocytes, and adipocytes (Pittenger et al., 1999; Robey et al., 2015). The regulatory mechanism of MSCs ontogeny has been extensively applied for clinical applications (Honmou et al., 2012; Chen et al., 2015; Forbes and Newsome, 2016; Haldar et al., 2016; Ji et al., 2017; Matthay et al., 2017). Stem cell fate determination is a process by which a multipotent stem cell or a progenitor cell is able to develop into one of multiple cell lineages followed by differentiation into mature cells. An important mechanism governing stem cell fate determination is epigenetic regulation that encompasses histone modifications (Zhu et al., 2013), DNA methylation, chromatin remodeling, specific transcriptional regulators (Hirabayashi and Gotoh, 2010), and microRNAs.

MicroRNAs (miRNAs) are small endogenous non-coding single-stranded RNA (18–25 nucleotides in length) (Rodriguez et al., 2004; Mourelatos, 2008) that regulates gene expression at the post-transcriptional level by binding to the 3′UTR region of the target mRNA (Lindsay, 2008). The pivotal role of miRNAs lies in its ability to mediate the negative regulation of gene expression by base pairing with complementary sequences. This pairing downregulates the expression of target genes by inducing mRNA degradation or translational inhibition at the post-transcriptional level (Hammond et al., 2001; Bartel, 2004; He and Hannon, 2004; Valencia-Sanchez et al., 2006; Winter et al., 2009). Previous studies had revealed that miR-9 and let-7b regulated the neural stem cell fate determination by targeting nuclear receptor TLX, which controls terminal differentiation into neurons (Zhao et al., 2009, 2010). Osteoblasts and adipocytes are two cell lineages that both originated from MSCs. The commitment of these two lineage is mutually unique and thus the understanding of this plasticity mechanism could pave the way for further understanding of osteoporosis or other bone diseases, which are caused by the infiltration of the bone tissue by adipocytes (Berendsen and Olsen, 2014).

During osteogenic differentiation from MSCs, regulatory transcription factors that are involved includes *RUNX2* (core binding factor α1) and *Osterix* (Shui et al., 2003; Tu et al., 2006; Choi et al., 2011; Zhang et al., 2011). In the meanwhile, the adipogenic differentiation from MSCs is mediated by *PPAR*γ (peroxisome proliferator-activated receptor gamma), which is a major regulatory transcription factor (David et al., 2007). It has been reported that there exists an inverse relationship between osteogenic and adipogenic differentiation in which the differentiation potential of rat marrow stromal cells (rMSCs) into adipocytes are inhibited whereas the differentiation potential of osteogenic cells are enhanced when rMSCs are cultured in dexamethasone and vitamin D3 (Beresford et al., 1992). Predominant fat cells presented in the bone marrow are characteristic of bone aging and osteoporosis. The decision of MSC differentiation into adipocyte is determined by cellular response mechanisms that involves an increase of adipogenic gene expression namely *PPARγ2* and the fatty acid binding protein *aP2*. Conversely, the expression of osteoblast markers (*RUNX2*, *Dlx5*, collagen, and osteocalcin) were found to be decreased (Moerman et al., 2004). However, the governing mechanism of this cell fate determination have not yet been elucidated. One plausible mechanism is that miRNAs specific to *PPAR*γ may give rise to mRNA degradation. In this study, bioinformatics tools were utilized for the prediction of miRNAs that are responsible for regulating osteogenic differentiation from human MSCs via the use of 3′UTR of *PPAR*γ gene and miRNA database. In the search for the candidate miRNA with specificity toward the *PPAR*γ gene, bioinformatics tools are considered as a rapid and cost-efficient approach (Stajich and Lapp, 2006). The candidate miRNA was identified by aligning the miRNA seed region to the mRNA sequence of target genes as the main prediction feature. Particularly, miR-130 had previously been reported to lower *PPAR*γ thereby resulting in the suppression of adipogenesis (Lee et al., 2011). Furthermore, miR-27 had previously been shown to play a key role in the reduction of adipogenic differentiation by targeting the *PPAR*γ (Karbiener et al., 2009; Lin et al., 2009; Kim et al., 2010). On the contrary, miR-27 has been shown to enhance the osteogenic differentiation via the activation of Wnt signaling by directly targeting and inhibiting the adenomatous polyposis coli (APC) gene (Wang and Xu, 2010). However, the role of miR-130a and miR-27b in human bone marrow MSCs fate decision through the regulation of *PPAR*γ gene expression has not yet been elucidated.

It is therefore the aim of this study to explore the role of miR-130a and miR-27b in the down-regulation of *PPAR*γ during osteogenesis of human MSCs *in vitro*. The expression of miR-130a and miR-27b were detected during the osteogenesis and adipogenesis of human bone marrow MSCs. Thus, an overexpression of miR-130a and miR-27b via lipofectamine transfection was performed in MSCs as to investigate their roles in osteogenesis. The understanding of how miRNA regulates *PPAR*γ during osteogenesis would provide great benefit for their clinical application for bone diseases.

## Materials and Methods

### Bioinformatics Method

**FIGURE 1:**
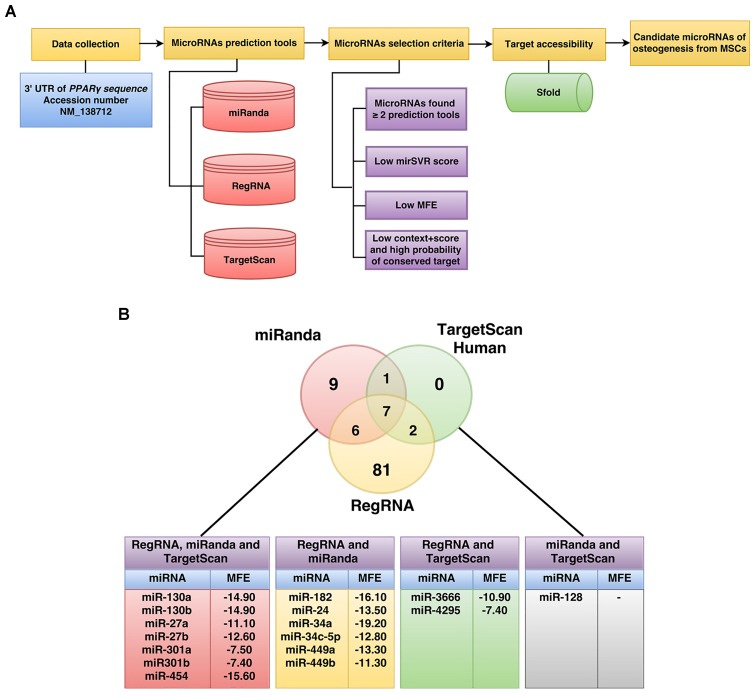
Schematic diagram of the workflow for the identification of miRNAs specific to the 3′UTR of *PPAR*γ **(A)**. The number of *PPAR*γ-specific miRNAs using RegRNA, miRanda, and TargetScanHuman are shown in the Venn’s diagram **(B)**. The miRNAs listed in the table below were identified by all three software or by two of the software are shown along with their corresponding MFE values **(B)**.

The workflow implemented for miRNA prediction in this study is summarized in Figure 1. The human 3′UTR sequence of *PPAR*γ was obtained from the NCBI database^1^ while predicted miRNAs were obtained from miRanda (Betel et al., 2010), RegRNA (Huang et al., 2006; Chang et al., 2013), and TargetScan (Lewis et al., 2005). Results of predicted miRNAs from the aforementioned softwares were subjected to a selection criteria to give rise to candidate miRNAs. Particularly, the candidate miRNA selection criteria is as follows: (1) exhibited greater than or equal to 2 in 3 prediction tools, (2) high negative free energy, (3) high negative mirSVR score, and (4) high negative context+score and high probability of conserved target (Seenprachawong et al., 2016). Candidate miRNAs fulfilling the selection criteria were further assessed in terms of their target accessibility using the Sfold software, which is a statistical sampling algorithm for predicting the RNA secondary structure that is accessible for RNA-targeting nucleic acids through base-pairing interactions (Ding and Lawrence, 2003; Ding et al., 2004).

### Mesenchymal Stem Cells Isolation and Culture

Human bone marrow from four healthy donors were collected in an aseptic condition from Ramathibodi Hospital with informed consent as part of the study protocol that was approved by the Faculty of Medicine, Ramathibodi Hospital, Mahidol University (MURA2017/603). Fresh human bone marrow was diluted with 1× PBS (ratio 1:1) and was gently layered on Histopaque 1.077 at a dilution of 1:1 in 15 ml centrifuge tube that is subsequently centrifuged at 400 × *g* for 30 min at 25°C. The PBMC layer was transferred to a new centrifuge tube and washed twice with 1× PBS. Cell pellet was resuspended with low-glucose Dulbecco’s Modified Eagle’s medium (DMEM-LG; GIBCO, United States) supplemented with 10% Fetal Bovine Serum (FBS; Merck Millipore, United States), 1% GlutaMAX (GIBCO, United States), and 1% Penicillin and Streptomycin (GIBCO, United States) and incubated at 37°C in a humidified atmosphere containing 95% air and 5% CO_2_. All experiments in this study were performed by MSCs passage 3–6.

### MSCs Characterization

Human MSCs were identified according to the ISCT criteria including morphology, cell surface markers and the capacity of differentiation.

#### Cell Surface Marker

Human MSCs were grown until confluence and cells between passages 3–6 were used for the characterization of human MSCs markers. In brief, human MSCs were trypsinized and wash with 1× PBS twice. Then, the number of cells were adjusted to 50,000 cells per tube. Cell surface markers on human MSCs were analyzed using a panel of antibodies against CD73 (PE-Cy7), CD90 (APC), CD105 (PE), CD34 (PE), and CD45 (PerCP). After the addition of the antibody, cells were incubated for 30 min at 4°C in the dark. Human MSCs were washed with 1× PBS twice at 4°C 2,000 rpm for 5 min. Cells were fixed with 1% paraformaldehyde 300 μl and analyzed with FACSCanto^TM^ II Flow Cytometry (BD Biosciences).

#### Osteogenic and Adipogenic Differentiation

Osteogenic differentiation of human MSCs were induced at approximately 80% confluence in osteogenic differentiation medium (ODM) containing 10% FBS complete DMEM-LG, 0.1 μM Dexamethasone, 10 mM β-Glycerophosphate and 50 μg/ml Ascorbic acid (All purchased from Sigma-Aldrich, St. Louis, MO, United States). ODM was changed every 3 days. Matrix mineralization or calcium depositions were examined with alizarin red S staining at day 21. Cells were observed by inverted microscopy.

Adipogenic differentiation of human MSCs were induced at approximately 80% confluence in adipogenic differentiation medium (ADM) containing 10% FBS complete DMEM-LG, 0.5 mM 3-Isobutyl-1-methylxanthine (IBMX), 1 μM Dexamethasone, 10 μM Insulin, and 200 μM Indomethacin (all purchased from Sigma-Aldrich, St. Louis, MO, United States). ADM was replaced every 3 days. Mature adipocytes or fat droplets formations were visualized by staining with Oil Red O solution. Cells were observed by inverted microscopy.

### MicroRNAs Transfection

For miRNAs transfection, human MSCs were plated at 1 day before transfection at a concentration such that cells could reach 80% confluence on the day of transfection. The functional role of miR-130a and miR-27b was verified by transfecting human MSCs with miR-130a mimic, miR-27b mimic, miR-130a inhibitor, miR-27b inhibitor, and its negative controls (Applied Biosystems/Ambion, Austin, TX, United States) using the Lipofectamine 3000 transfection agent (Invitrogen, Carlsbad, CA, United States) according to the manufacturer’s instructions. After cells were cultured in the medium for 3 days, the efficiency of miRNAs transfection was determined by RT-qPCR. At the indicated time points, cells were harvested for miRNA expression, mRNA expression, alkaline phosphatase activity and protein analysis.

### RNA Extraction and Reverse-Transcription Quantitative Polymerase Chain Reaction

Total RNA was extracted with TRIzol (Invitrogen, Carlsbad, CA, United States) at 0, 3, 7, 10, and 14 days after induction for quantitative real-time PCR analysis. Total RNA was purified by using Direct-zol RNA Mini Prep (Zymo Research) according to the manufacturer’s protocols. Aliquots of cDNAs were amplified with primers for *RUNX2*, *Osterix*, *PPAR*γ, *C/EBP*β, miR-130a and miR-27b (Primer sequences in Supplementary Tables S1, S2).

Quantification of miRNAs were performed using either the TaqMan method with the U6 snRNA as an internal control (TaqMan MicroRNA Reverse Transcription Kit and TaqMan Universal PCR Master Mix; Applied Biosystems/Ambion, Austin, TX, United States), according to the manufacturer’s protocols.

Expression levels of mRNA were quantified in total RNA using the SYBR Green method with endogenous gene *GAPDH* as control for normalization. Real-time PCR was performed in CFX96^TM^ Real-Time PCR Detection System (Bio-Rad). Gene expressions were amplified by PCR for 40 cycles with each cycle at 95°C for 3 s, 60°C for 30 s, and 72°C for 20 s.

### Western Blot Analysis

Whole-cell lysates were prepared on ice using 0.5 ml cold RIPA lysis buffer (Merck Millipore) containing protease inhibitor (Thermo Fisher Scientific). In brief, equal amounts of proteins (30 μg) were separated by sodium dodecyl sulfate–polyacrylamide gel electrophoresis (SDS–PAGE) and transferred to nitro cellulose membranes. After incubation with monoclonal mouse anti-PPARγ (1:250 diluted; Santa Cruz Biotechnology, CA, United States), anti-RUNX2 (1:1000 diluted; Santa Cruz Biotechnology, CA, United States), anti-COL1A1 (1:2000 diluted; Chemicon, Merck Millipore), anti-Osterix (1:1000 diluted; Abcam) and β-actin (1:5000 diluted, Chemicon, Merck Millipore) at 4°C overnight, they were further immunoblotted with HRP-conjugated horse anti-mouse IgG antibody (1:10000 diluted; Cell Signaling, United States) at 37°C for 90 min, developed with enhanced chemiluminescence (ECL) substrate (Amersham ECL Prime Western Blotting Detection Reagent, GE Healthcare) and chemiluminescence detection by ChemiDoc^TM^ MP Imaging System (Bio-Rad, United Kingdom). Band density was quantitated using the Image Lab^TM^ software Version 5.2.1 (Bio-Rad, United Kingdom).

### ALP Activity

Human MSCs cultured in ODM were transfected with miR-130a mimics or inhibitor, miR-27b mimics or inhibitor and its negative controls (Applied Biosystems/Ambion, Austin, TX, United States) using the Lipofectamine 3000 transfection agent (Invitrogen, Carlsbad, CA, United States). The alkaline phosphatase activity was measured using an assay kit (Colorimetric; Abcam). At indicated times, the conditioned medium was collected. Into a 96-well plate, 80 μl of samples was added followed by the addition of 50 μl of a 5 mM pNPP solution to each well. The reaction mixture was incubated at 25°C for 60 min and the reaction was stopped by adding 20 μl of the stop solution followed by gently shaking of the plate. The *p*-nitrophenol product, which was generated via enzymatic hydrolysis of the *p*-nitrophenylphosphate substrate, was detected at OD 405 nm using a microplate reader synergy HTX Multi-Mode Reader (BioTek Instruments, VT, United States).

### Immunofluorescence Staining

For osteogenic and adipogenic protein expression, MSCs were cultured on coverslips with ODM and ADM for 14 days. At day 7, 10, and 14, cells were fixed with 4% paraformaldehyde in PBS, followed by washing with PBS twice. Cells were permeabilized with 0.3% Triton X-100 (Merck KGaA) in PBS, non-specifically blocked with 3% BSA (Thermo Fisher Scientific, Inc.) in PBS, and incubated with mouse anti-RUNX2 antibodies and mouse anti-PPARγ antibodies (1:50; Santa Cruz Biotechnology, CA, United States) at 4°C overnight. After three washes with PBST, the cells were incubated with goat anti-mouse IgG (1:500; H+L; Alexa Fluor 488; Abcam) for 1 h at room temperature and then counterstained with antifade containing DAPI (Invitrogen; Thermo Fisher Scientific, Inc.). The fluorescent images were obtained with a confocal laser scanning microscope and analyzed with FluoView FV1000 Software version 3.01 (Olympus Corp., Tokyo, Japan).

### Statistical Analysis

All data are presented as the mean ± standard error of the mean (SEM) from tripicate experiments. Statistical analysis was performed using the Mann-Whitney *U*-test via the PASW Statistics, version 18. A *p-*value of <0.05 was used as the threshold of statistical significance.

## Results

### Computational Identification of PPARγ Specific miRNAs During Osteogenic Differentiation From Human Mesenchymal Stem Cells

To identify potential miRNAs with specificity toward *PPAR*γ, we computationally identified miRNAs using three bioinformatics tools namely RegRNA^2^, TargetScan^3^, and miRanda^4^. Four steps of prediction were applied to identify candidate miRNAs as shown in the schematic diagram of the workflow (Figure 1A). Initially, the 3′UTR sequence of *PPAR*γ (NM_138712) was retrieved from the NCBI database. Next, sequences were submitted to RegRNA, TargetScan and miRanda tools which resulted in the selection of 96, 10, and 23 predicted miRNA (Figure 1B and Supplementary Data 1). The predicted miRNAs were again chosen according to the selection criteria and only 16 miRNA with complete match were categorized as candidate miRNAs (Supplementary Data 2). The candidate miRNAs were evaluated for mRNA accessibility using the Sfold software and 9 miRNAs (miR-454, 130a, 130b, 27a, 27b, 301a, 301b, 3666, and 4295) afforded probability of more than 0.5. The resulting candidate miRNAs were then reviewed for their relevance and role in stem cell research in which only miR-130a and miR-27b were selected for further characterization.

### Human MSCs Characterization

**FIGURE 2:**
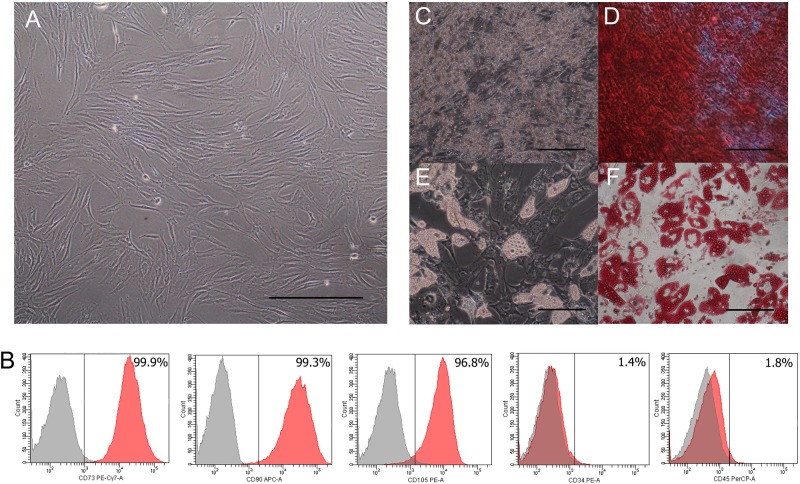
Osteogenic and adipogenic differentiation capacity from human bone marrow MSCs. Morphology (Scale bar = 500 μm) **(A)** and flow cytometric analysis **(B)** of human bone marrow MSCs. Human MSCs from the bone marrow were subjected to characterization and they were found to be positive (≥95%) for CD73, CD90, and CD105 and negative (≤2%) for hematopoietic lineage markers CD34 and CD45. Human MSCs from the bone marrow were cultured in an osteogenic differentiation medium for 21 days where an accumulation of the matrix and calcium deposition **(C)** were observed and positive staining with alizarin red S **(D)** was carried out. The adipogenic differentiation of human MSCs were performed by culturing them in an adipogenic differentiation medium. After 21 days, they were shown to have round-shaped cells containing lipid droplets in the cytoplasm **(E)**. Results of oil red O staining (cytochemical staining for fat droplets) showed positive staining **(F)**. Scale bars of panels **C–F** = 50 μm.

Human MSCs were characterized according to the ISCT criteria that encompasses morphology, cell surface markers, and capacity of differentiation. Human MSCs had a fibroblast-liked morphology and could adhere to the plastic culture flask after 3 days of culture (Figure 2A). Human MSCs were shown to express approximately 95% of CD73, CD90, and CD105 positive cells. In contrast, they were found to be positive (≤2%) for hematopoietic lineage markers CD34 and CD45 (Figure 2B). The characterization of human MSCs based on the mesodermal differentiation capability was performed in which human MSCs were cultured in an ODM. After 21 days, they were shown to afford matrix accumulation, calcium deposition (Figure 2C) and positive staining with alizarin red S (Figure 2D). As for the adipogenic differentiation, human MSCs were cultured in an ADM. After 21 days, large round cells containing numerous lipid droplets in the cytoplasm were observed (Figure 2E). The oil red O staining was used to characterize adipocyte liked-cells. Results showed positive staining of fat droplets in the cytoplasm (Figure 2F).

### Osteogenic and Adipogenic Gene and Protein Expression During *in vitro* Differentiation

**FIGURE 3:**
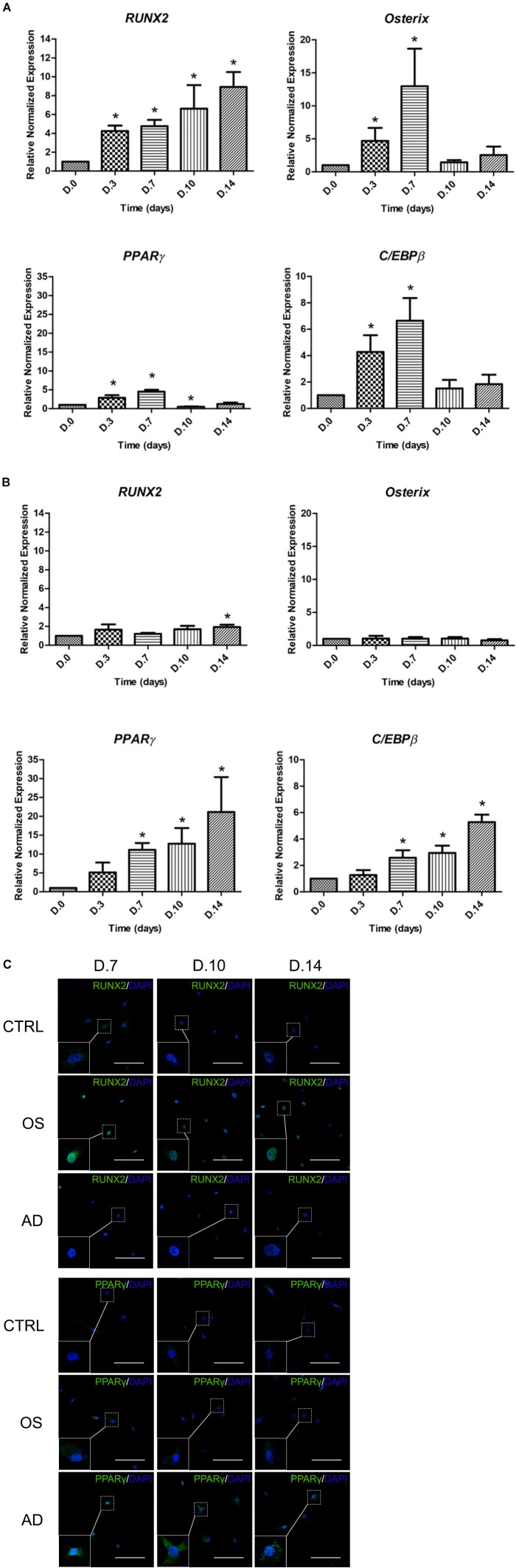
The Expression of osteogenic-specific markers (*RUNX2*, *Osterix*) and adipogenic-specific markers (*PPAR*γ, *C/EBP*β) during osteogenic differentiation **(A)** and adipogenic differentiation **(B)**. Total RNA was prepared at indicated times and subjected to quantitative real-time PCR analysis. The mRNA expression was normalized to the levels of *GAPDH* mRNA. The data shown are the average from four independent experiments (mean value ± SEM). ^∗^*p-*value < 0.05 was detected when compared with day 0. Immunofluorescent study of RUNX2 and PPARγ **(C)** during osteogenic (OS) or adipogenic (AD) differentiation and control (CTRL) at days 7–14. Scale bar = 200 μm.

The expression of master transcription factors of osteogenesis (*RUNX2* and *Osterix*) and adipogenesis (*PPAR*γ and *C/EBP*β) were investigated in MSCs cultured with ODM and ADM, respectively, at days 0, 3, 7, 10, and 14 (Figure 3). Results demonstrated a significant increase in the expression of *RUNX2* since day 0 until day 14 while in *Osterix* it was detected since day 0 until day 7 in MSCs with osteogenic differentiation. Simultaneously, the expression of *PPAR*γ and *C/EBP*β were found to be significantly increased since day 0 until day 7 while a decreasing trend was observed since day 10 until day 14 (Figure 3A). In MSCs cultured with the ADM, no difference were detected in the expression of *RUNX2* and *Osterix* from days 0–14. In the meanwhile, a gradual increase of *PPAR*γ and *C/EBP*β was observed since days 0–14 with significant difference (*p* < 0.05) (Figure 3B). The fluorescent study for the expression of RUNX2 and PPARγ were then performed. The presence of RUNX2 was shown from days 7 to 14 and localized in nucleus of MSCs cultured with ODM (Figure 3C), whereas PPARγ showed weak positive. The presence of PPARγ were observed in MSCs cultured with the ADM from days 7 to 14, whereas a lower intensity was found in MSCs cultured with the ODM. The localization of PPARγ was distributed in the nucleus and cytoplasm (Figure 3C).

### Expression of miRNAs During Osteogenic and Adipogenic Differentiation

**FIGURE 4:**
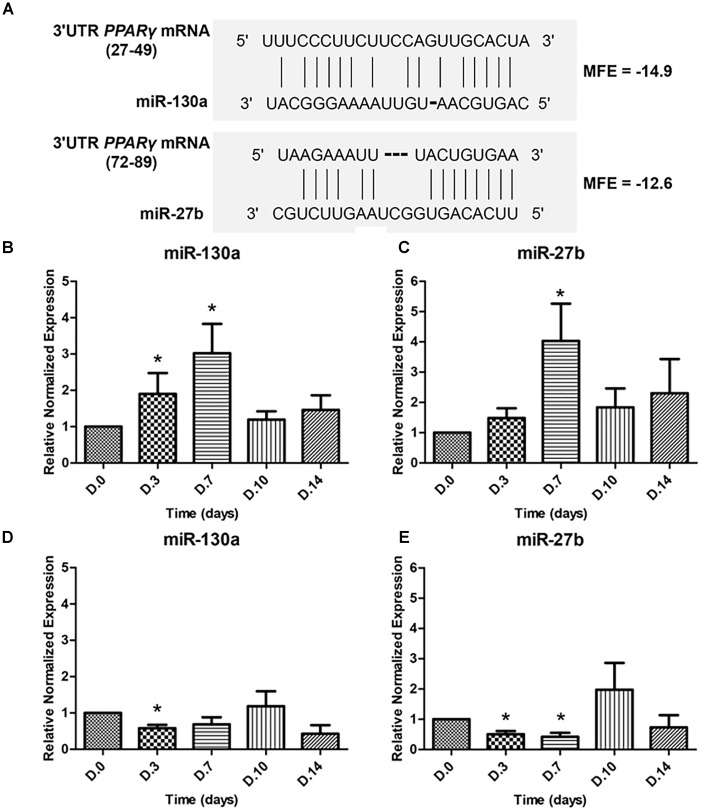
Duplex structure of miRNAs and 3′UTR *PPAR*γ, minimal free energy (MFE) and the pairing position **(A)**. Expression of miR-130a (**B**) and miR-27b (**C**) during osteogenic differentiation and expression of miR-130a **(D)** and miR-27b **(E)** during adipogenic differentiation. Expression of miR-130a and miR-27b were quantitatively assessed by TaqMan probe-based quantitative real-time PCR. The miRNAs expression level was normalized to levels of U6snRNA. The data shown are average of four independent experiments (mean value ± SEM). ^∗^*p-*value < 0.05 as compared with day 0.

To determine whether miR-130a and miR-27b controls the activity of *PPAR*γ or not, the ability of the 3′UTR of human *PPAR*γ to bind miR-130a and miR-27b was confirmed by the fact that all three prediction tools exhibited high negative values in the free energy, which suggested plausible hybridizations of miRNA and target mRNA duplex (Figure 4A). The expression of miR-130a and miR-27b were evaluated in MSCs cultured with ODM and ADM, respectively, at days 0, 3, 7, 10, and 14 (Figures 4B–E). Results revealed that there was a significant increase in the expression of miR-130a and miR-27b from day 0 until day 7 during osteogenic differentiation while a decreasing trend was observed since day 10 until day 14 (Figures 4B,C). In contrast, the expression of miR-130a and miR-27b showed a significantly decreased expression since day 0 until day 7 for adipogenic differentiation (Figures 4D,E).

### The Expression Level of miR-130a and miR-27b After Transfection

**FIGURE 5:**
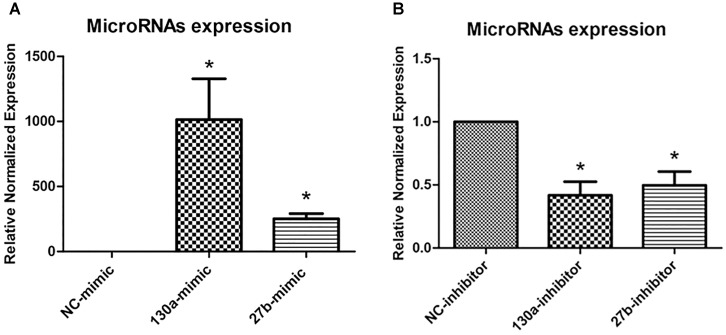
Expression of mimics **(A)** and inhibitors **(B)** of miR-130a and miR-27b after 3 days of transfection were quantitatively assessed using the TaqMan probe-based quantitative real-time PCR. The miRNAs expression was normalized to the levels of U6snRNA. The data shown are the average from three independent experiments (mean value ± SEM). ^∗^*p*-value < 0.05 as compared with negative control.

To investigate the roles of miR-130a and miR-27b in osteogenesis, we transiently transfected human MSCs with miR-130a mimic, miR-27b mimic, miR-130a inhibitor, and miR-27b inhibitor. After 3 days of transfection, the expression level of miR-130a and miR-27b were analyzed as presented in Figure 5. It was observed that there were significantly increased expression of miR-130a and miR-27b in miR-mimic condition (Figure 5A). Conversely, the expression of miR-130a and miR-27b in the presence of miR-inhibitor were shown to be significantly decreased (Figure 5B).

### Peroxisome Proliferator-Activated Receptor γ (PPARγ) Expression in Human MSCs After Transfection With Target miR-130a and miR-27b Mimic and Inhibitor

**FIGURE 6:**
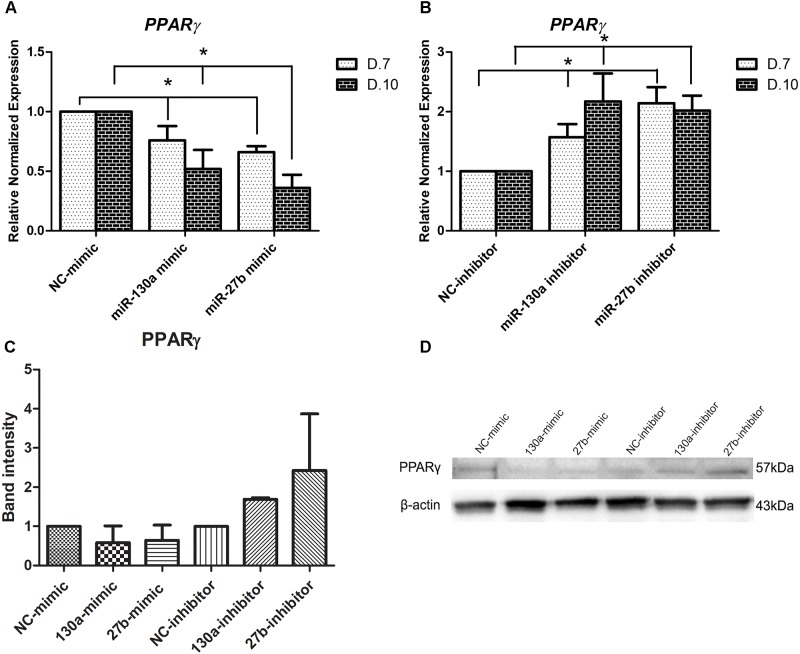
**(A,B)** Expression of *PPAR*γ in human MSCs transfected with mimics and inhibitors of miR-130a and miR-27b at days 7 and 10. Results of *PPAR*γ expression was normalized to levels of *GAPDH* mRNA. The data shown are average of three independent experiments (mean value ± SEM). ^∗^*p-*value < 0.05 were compared with the negative control. **(C,D)** Western blot analysis of the expression of PPARγ in human MSCs transfected with mimics and inhibitors of miR-130a and miR-27b along with their negative control. Analysis was performed at day 14 and images were quantified by the ImageJ software. β-actin served as the loading control (40 μg protein loaded; *n* = 2; mean value ± SEM).

To explore whether *PPAR*γ can be down-regulated by miR-130a and miR-27b in human MSCs during osteogenesis, we performed transient transfection with mimics and inhibitors of miR-130a and miR-27b in MSCs cultured with the ODM. *PPAR*γ gene expression was detected at days 7 and 10. Results showed that there were significantly decreased expression of *PPAR*γ for MSCs transfected with mimics of miR-130a and miR-27b (*p* < 0.05) (Figure 6A). In the meanwhile, the expression of *PPAR*γ in MSCs transfected with inhibitors of miR-130a and miR-27b were shown to be significantly increased (*p* < 0.05) (Figure 6B). Western blot analysis of PPARγ was investigated and presented by the mean and SEM values as shown in the bar graph (Figure 6C). The protein band for PPARγ of MSCs transfected with miR-130a and miR-27b mimic showed lower intensity than that of the negative control. MSCs transfected with the inhibitor of miR-130a and miR-27b showed higher intensity for PPARγ than the negative control (Figure 6D). These data indicated that *PPAR*γ is the direct target of miR-130a and miR-27b in human MSCs.

### The Effect of miR-130a, miR-27b in the Osteogenesis of Human MSCs

#### Quantitative RT-PCR of RUNX2 and Osterix

**FIGURE 7:**
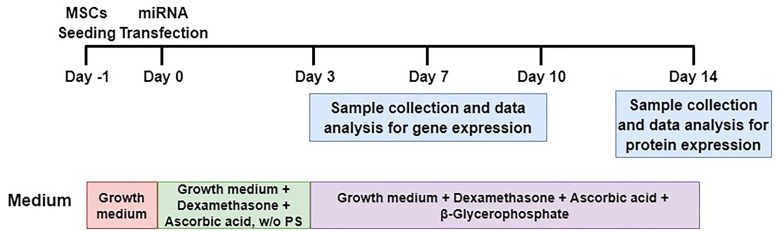
Schematic diagram of MSCs transfection and osteogenic differentiation model. MSCs were transfected with miRNAs and their anti-miRNAs (day 0). Differentiated cells (days 3, 7, and 10) were collected for analyses of mRNA levels and differentiated cells at day 14 were collected for protein expression. It is worthy to note that w/o PS means without Penicillin and Streptomycin.

**FIGURE 8:**
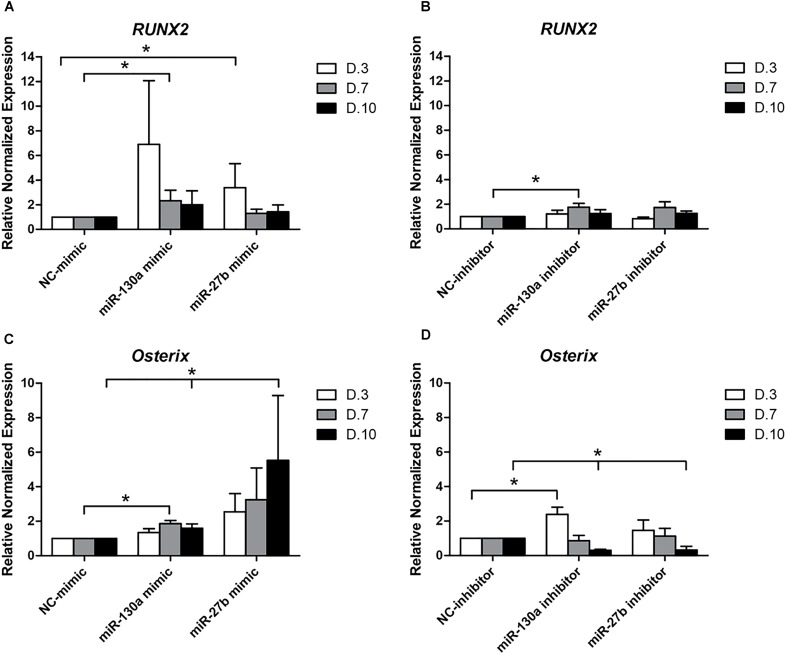
Expression of *RUNX2* **(A,B)** and *Osterix* **(C,D)** at days 3, 7, and 10 after transfecting with mimics and inhibitors of miR-130a and miR-27b. Total RNA was prepared at indicated times and subjected to quantitative real-time PCR analysis. The expression of *RUNX2* and *Osterix* were normalized to the level of *GAPDH* mRNA. The data shown are average of three independent experiments (mean value ± SEM). ^∗^*p-*value < 0.05 as compared with negative control in each day.

To elucidate whether or not the overexpression and reduction of miR-130a and miR-27b levels exert any influence on the osteogenic differentiation or not, mimics and inhibitors of miR-130a and miR-27b were transiently transfected in human MSCs. Transfected cells were grown in an ODM and the expression of osteogenic-specific genes (*RUNX2, Osterix*) were investigated at days 3, 7, and 10 while protein analysis was carried out at day 14 (Figure 7). The expression results showed an increased expression of *RUNX2* at day 3 and gradually decreased from days 7 to 10 (Figure 8A) in miR-130a and miR-27b mimic transfected MSCs. The expression of *Osterix* was gradually increased from days 3 to 10 after miR-130a and miR-27b mimic transfection (Figure 8C). MSCs transfected with miR-130a and miR-27b inhibitors showed the similar expression of *RUNX2* to that of the control whereas the expression of *Osterix* was gradually decreased from days 3 to 10 (Figures 8B,D). Collectively, these results indicated that the overexpression of miR-130a and miR-27b promotes osteogenic differentiation by facilitating high expression of *RUNX2* and *Osterix*. The expression levels of osteogenic-specific genes (*RUNX2*, *Osterix*) and *p*-value were shown in Supplementary Data 3 (Supplementary Table S1).

#### Western Blot Analysis for COL1A1, RUNX2, Osterix

**FIGURE 9:**
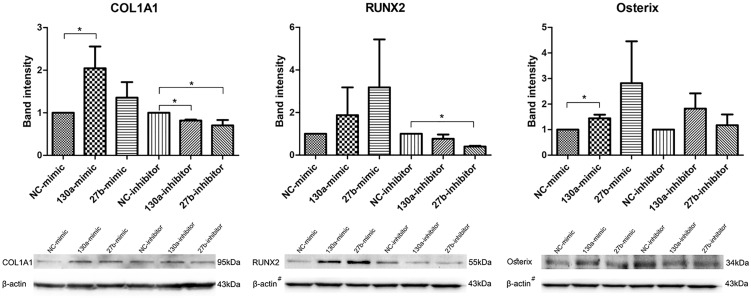
Western blot analysis of the expression of COL1A1, RUNX2, and Osterix in human MSCs transfected with mimics and inhibitors of miR-130a and miR-27b along with their negative control. Analysis was performed for 14 days and images were quantified by the ImageJ software. β-actin served as the loading control. The data shown are average of three independent experiments (mean value ± SEM). ^∗^*p-*value < 0.05 as compared with the negative control. ^#^The β-actin control in Western blot result for RUNX2 and Osterix was from the same membrane.

Western blot analysis of COL1A1, RUNX2 and Osterix were investigated and quantitatively studied. The densitometric analysis of the protein intensity were presented by the mean and SEM values as shown in the bar graph (Figure 9). The protein band of MSCs transfected with miR-130a and miR-27b mimic, which showed higher intensity than that of the control. MSCs transfected with the inhibitor of miR-130a and miR-27b showed significantly lower intensity for COL1A1 and RUNX2 as compared to the control and no significant difference was observed for Osterix (Figure 9). Statistical tests were applied as to compare treatments (mimic or inhibitor) and control (NC) for each markers. In MSCs transfected with the miR-130a mimic, *p-*value of COL1A1, RUNX2, and Osterix were 0.037, 0.487, and 0.037, respectively. In MSCs transfected with the miR-130a inhibitor, *p-*value of COL1A1, RUNX2, and Osterix were 0.037, 0.487, and 0.487, respectively. In MSCs transfected with miR-27b mimic, the *p-*values of COL1A1, RUNX2, and Osterix were 0.487, 0.487, and 0.487, respectively. And MSCs transfected with miR-27b inhibitor, *p-*value of COL1A1, RUNX2, and Osterix were 0.037, 0.037, and 0.487, respectively.

#### Alkaline Phosphatase Activity and Alizarin Red S Staining

**FIGURE 10:**
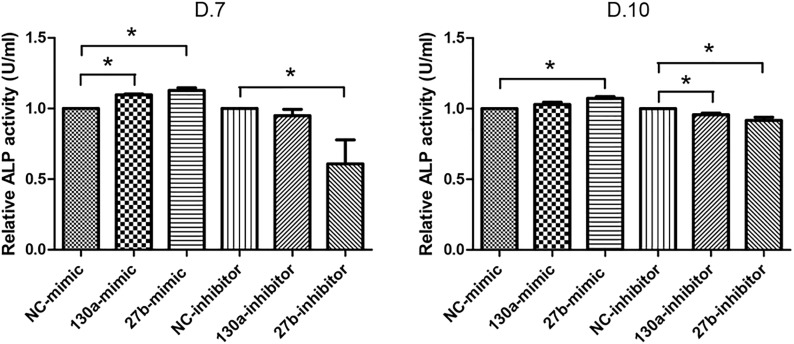
Analysis of alkaline phosphatase (ALP) activity in conditioned medium. Shown are the activity (U) per milliliter (ml) of human MSCs transfected with mimics and inhibitors of miR-130a and miR-27b along with its negative control after 7 and 10 days of transfection. The data shown are average of three independent experiments (mean value ± SEM). ^∗^*p-*value < 0.05 as compared with the negative control.

**FIGURE 11:**
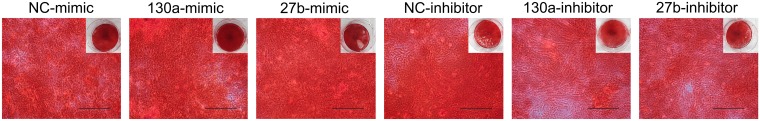
Staining of calcium deposition by alizarin red S in human MSCs after treatment with mimics and inhibitors of miR-130a and miR-27b along with their negative control under osteogenic differentiation medium for 21 days. Scale bars = 500 μm.

Results from alkaline phosphatase activity and alizarin red S staining supported the mRNA and protein results as shown in Figures 10,11. Particularly, the effects of mimics of miR-130a and miR-27b on the ALP activity was shown to be significantly increased at days 7 and 10 in comparison to that of the negative control but was found to be significantly decreased in those transfected with inhibitors of miR-130a and miR-27b (Figure 10). The functional assay was also confirmed by alizarin red S staining in human MSCs after treatment with mimics and inhibitors of miR-130a and miR-27b along with their negative control under ODM for 21 days. An increased presence of calcium deposits was found within the mimics of miR-130a and miR-27b but decreased presence of calcium deposits was found within the inhibitors of miR-130a and miR-27b when compare with their negative control (Figure 11). Taken together, these data suggested that miR-130a and miR-27b enhanced osteogenic differentiation of human MSCs.

## Discussion

Mesenchymal stem cells inherently possesses the potential of differentiating into multi-cell types that originates from the mesodermal germ layer. The prominent function of MSCs is to maintain homeostasis for balancing organ function. The disturbance of MSCs growth and differentiation was revealed to play a key role in the pathologic organ dysfunction and the underlying cause was largely unknown. A plausible explanation may be the resulting imbalanced levels of stem cells that arises from such delineation that leads to the replacement of bone tissues with adipose tissues as found in many pathological bone diseases such as osteoporosis and osteopenia. Both osteoblast and adipocyte share a common progenitor, which are the MSCs. The determination of MSCs in osteogenesis and adipogenesis was strictly regulated by external factors (i.e., extracellular matrix, growth factor, and cytokine) and internal factors. In regards to internal factors, it has been vastly reported (Komori, 2006; Lefterova et al., 2008; Augello and De Bari, 2010; Perez-Campo and Riancho, 2015) that specific lineage transcription factors, signaling molecules, and epigenetic machinery governs cell fate determination. Recently, miRNA has been mentioned to be epigenetic factors governing RNA silencing and post-transcriptional regulation of the gene expression. The role of miRNA in stem cells delineation was reported to be based on their roles in targeting specific mRNAs for the repression of degradation or translation, which represents key mechanisms for cell differentiation, growth, mobility and apoptosis. The roles of miRNA in osteogenic and adipogenic differentiation were dispersedly reported (Martin et al., 2016; Huang et al., 2017; Zaiou et al., 2018) and a clear osteoblast-adipocyte differentiation relationship could not be made. Our study sheds light on the novel roles of miRNA specific to *PPAR*γ and specific transcription factor for adipogenesis in MSCs during osteogenesis.

It was found that the expression of *RUNX2* and *Osterix* (i.e., key transcription factor of osteogenesis) are highly expressed in MSCs whereas *PPAR*γ and *C/EBP*β (i.e., key transcription factor of adipogenesis) are decreased at day 10 of *in vitro* osteogenesis. This expression pattern was in correspondence with that of a previous study in the fate decision of MSCs to specific osteogenic lineage (Post et al., 2008; Taipaleenmaki et al., 2011). The mechanism behind this event could be ascribed to the posttranscription control of the *PPAR*γ expression by miRNAs. Currently, many methods have been proposed to explore the miRNA function such as microarrays and direct cloning. However, these approaches are cost and time consuming as well as difficulty in data interpretation (Tome et al., 2011; Huang et al., 2012). Bioinformatics tools are powerful tools for investigating of genes, proteins, mRNA, and miRNA profiles. For the recent few decades, computational tools have been implemented to identify novel miRNA-disease associations. Especially, the miRNA-disease associations have been experimentally confirmed (Fallah et al., 2015). Identifying disease-related miRNAs has become an important goal of biomedical research, which will accelerate the understanding of the pathological mechanisms underlying complex human diseases. To identify the potential miRNAs of *PPAR*γ, three software including RegRNA, TargetScan and miRanda were selected due to their user-friendly interface, easy to use, and provides good graphical visualization (Huang et al., 2006; Chang et al., 2013). The combination of multiple bioinformatics tools provided the beneficial information by reducing the chances of false positive results and creating high accuracy and precision data (Hamam et al., 2014; Cao et al., 2015; Li et al., 2016). The candidate miRNA generated from bioinformatics tools in our study were miR-454, 130a, 130b, 27a, 27b, 301a, 301b, 3666, and 4295. These candidate miRNAs have previously been reported to be associated with a number of human diseases (Shi et al., 2011; Wang et al., 2016; Zhu et al., 2016). However, the role of these candidate miRNAs in regulating osteogenic differentiation have not been investigated yet.

Many miRNAs specific to *PPAR*γ were discovered and reported to exert roles in health and pathologic conditions. However, its role in osteogenesis is not notable. Most studies on miRNAs that are specific to *PPAR*γ were focused on the adipogenesis process. Particularly, miR-27b has been shown to be able to post-transcriptionally affect the expression of *PPAR*γ and *C/EBP*α during the early stages of adipogenesis of human adiposed stem cells (Karbiener et al., 2009). Lin et al. (2009) have shown that miR-27 is a new class of adipogenic inhibitors as it could inhibit *PPAR*γ and may be involved in the development of obesity. In addition, miR-130 are known to influence the adipogenesis during the differentiation of human preadipocytes into adipocytes. Particularly, the overexpression and reduction of miR-130 leads to impair and enhance adipogenesis, respectively (Lee et al., 2011). These findings suggested that miR-130 and miR-27 act as negative regulators of adipogenesis by targeting *PPAR*γ. We investigated the expression level of miR-130a and miR-27b during *in vitro* osteogenesis and adipogenesis in which high expression of both miRNA were observed in osteogenesis while the expression of miR-130a and miR-27b were found to be low in adipogenesis. Therefore, we hypothesized that miR-130 and miR-27 might play an inverse role in influencing the fate decision of MSCs by modulating the *PPAR*γ activity in specifying the fate of MSCs to osteoblasts.

The specific cell type differentiation is a tightly controlled process that involves an intricate network of transcription factors acting at different time points during differentiation. Several studies have clearly established PPARγ as a key regulator which highly expressed in early stage of adipogenesis (Jeong et al., 2014; Nakamura et al., 2014). As a proof of concept, we performed the transient transfection of miR-130a and miR-27b mimic and inhibitor into human MSCs during osteogenic differentiation. Generally, the miRNA level from transient transfection persist 1–7 days which is in the phase of early differentiation stage. The expression of *PPAR*γ showed a decreased and increased after transfection with miR-130a and miR-27b mimic and inhibitor, respectively, thereby confirming that *PPAR*γ was indeed the target of miR-130a and miR-27b. However, the Western blot results of PPARγ, which was analyzed at day 14, revealed slight differences in the band intensity amongst the samples. This might suggest that such transient transfection may reduce their inhibitory property followed by recovery of the expression of PPARγ. Supplementing miR-130a and miR-27b activity through miR-130a and miR-27b mimics in human MSCs could promote osteogenesis via increased gene expression of *RUNX2* and *Osterix*, the protein expression of RUNX2, COL1A1 and Osterix, the alkaline phosphatase activity as well as that of alizarin red S staining. Conversely, the knockdown of miR-130a and miR-27b using specific inhibitors were shown to block the differentiation of osteoblasts from MSCs. In addition, this study represents a pilot study which imply the plausible mechanism of miRNA in MSCs fate determination using lipofectamine transfection. The process of osteogenic differentiation from MSC into osteoblast is a complex process that is controlled by a number of miRNAs and several factors. To clearly understand this process, further experimental study is still needed.

Thus, our results provides a novel mechanistic insight into how miRNAs mediated MSCs fate decision toward the phenotypes of osteoblasts. Herein, we report a direct link between miRNA properties and early MSC fate specification events. Owing to the complexity of osteogenesis in which it is regulated by miRNAs where one miRNA may have several target sites in the 3′UTR of a single mRNA, and one 3′UTR may have several different binding sites for different miRNAs. Therefore, multiple co-expression of miRNAs would thereby act in concert to repress the expressional level of a specific mRNA that finally results in osteogenic lineage commitment from MSCs. The alteration of the miRNAs expression pattern during adipogenesis and osteogenesis is largely unknown and have yet to be explored. Findings of this mechanism could be beneficial in the improvement of the quality of life in orthopedic patient. Currently, miRNAs have emerged as crucial modulators of osteoporosis. The circulating miRNAs have been identified as biomarkers for a postmenopausal osteoporosis, miR-133a was significantly upregulated in serum of osteoporotic women (Li et al., 2018). Several miRNAs have been identified in patients with osteoporosis by regulating the function of osteoblasts and osteoclasts (Taipaleenmaki, 2018). The knowledge gained from this study may lead to clinical applications encompassing early diseases diagnosis, prognosis as well as therapeutic applications. Therapeutic miRNAs by means of miRNA antagonists or miRNA mimics may be used to inhibit endogenous miRNA that may ultimately promote the osteogenesis of resident MSCs instead of adipogenesis. However, these findings have been obtained from *in vitro* studies and in order to move a step closer toward clinical applications, investigations involving *in vivo* models are also required.

## Ethics Statement

This study was carried out in accordance with the recommendations of Study of mesenchymal stem cells differentiation to osteocyte, chondrocyte, and tenocyte derived from bone marrow, adipose tissue, and umbilical cord (MURA2017/603), the Faculty of Medicine, Ramathibodi Hospital, Mahidol University. The protocol was approved by the Ramathibodi Hospital with informed consent as a part of the study protocol approved by the Faculty of Medicine, Ramathibodi Hospital, Mahidol University. All subjects gave written informed consent in accordance with the Declaration of Helsinki.

## Author Contributions

KS designed the study, collected the data, and performed the analysis. AS designed the studies. TT, CN, SH, and AS contributed to the discussion of the data. KS, PN, and AS contributed to the analysis of the data. KS, CN, and AS contributed to manuscript preparation. All authors read, critically discussed, and approved the final manuscript.

## Conflict of Interest Statement

The authors declare that the research was conducted in the absence of any commercial or financial relationships that could be construed as a potential conflict of interest.

## Footnotes

1 http://www.ncbi.nlm.nih.gov

2 http://regrna2.mbc.nctu.edu.tw

3 http://www.targetscan.org

4 http://www.microrna.org
