# Supplementary material for: miR-130a and miR-27b Enhance Osteogenesis in Human Bone Marrow Mesenchymal Stem Cells via Specific Down-Regulation of Peroxisome Proliferator-Activated Receptor γ
Source: Front Genet. 2018 Nov 14;9:543. doi: 10.3389/fgene.2018.00543 (PMC6246628; doi:10.3389/fgene.2018.00543)
Supplement: Supplementary file 1 [file Table_1.DOCX]

Supplementary Material

**miR-130a and miR-27b enhance osteogenesis in human bone marrow mesenchymal stem cells via specific down-regulation of peroxisome proliferator-activated receptor γ**

**Kanokwan Seenprachawong, Tulyapruek Tawornsawutruk, Chanin Nantasenamat, Pornlada Nuchnoi, Suradej Hongeng and Aungkura Supokawej^*^**

*** Correspondence:** Corresponding Author: Aungkura Supokawej, aungkura.jer@mahidol.ac.th

# Supplementary Data

List of predicted miRNAs binding to 3’UTR of *PPARγ* gene, which were predicted by computational tools including RegRNA V2.0, TargetScanHuman V6.2, and miRanda.

| **RegRNA**  **96 miRNAs** | **TargetScan**  **10 miRNAs** | **miRanda**  **23 miRNAs** |
| --- | --- | --- |
| miR-106a-5p, miR-106b-5p, miR-130a-3p, miR-130b-3p, | miR-128 | miR-101 |
| miR-142-5p, miR-144-3p, miR-17-5p, miR-182-5p, | miR-130a | miR-1271 |
| miR-206, miR-20a-5p, miR-20b-5p, miR-24-3p, | miR-130b | miR-128 |
| miR-2682-5p, miR-27a-3p, miR-27b-3p, miR-301a-3p, | miR-27a | miR-130a |
| miR-301b, miR-302c-3p, miR-30c-1-3p, miR-30c-2-3p, | miR-27b | miR-130b |
| miR-3121-3p, miR-3154, miR-3163, miR-3173-3p, | miR-301a | miR-144 |
| miR-3185,miR-3192, miR-33a-3p, miR-338-5p, | miR-301b | miR-153 |
| miR-34a-5p,miR-34a-3p, miR-34c-5p, miR-3664-3p, | miR-3666 | miR-182 |
| miR-3666, miR-3669, miR-373-3p miR-374a-3p, | miR-4295 | miR-24 |
| miR-409-3p, miR-424-5p, miR-4295, miR-4307, | miR-454 | miR-27a |
| miR-4446-5p, miR-449a, miR-449b-5p, miR-4519, |  | miR-27b |
| miR-454-3p, miR-4645-3p, miR-4662a-3p, miR-4671-3p, |  | miR-301a |
| miR-4703-5p, miR-4711-3p, miR-4781-3p, miR-5093, |  | miR-301b |
| miR-511, miR-513a-3p, miR-520b, miR-520c-3p, |  | miR-34a |
| miR-520d-3p, miR-520f, miR-526b-3p, miR-545-5p, |  | miR-34c-5p |
| miR-548a-5p, miR-548b-5p, miR-548c-5p, miR-548d-5p, |  | miR-340 |
| miR-548d-3p, miR-548h-5p, miR-548i, miR-548j, |  | miR-361-5p |
| miR-548m, miR-548n, miR-548o-5p, miR-548w, |  | miR-448 |
| miR-548y, miR-548ab, miR-548ag, miR-548ah-5p, |  | miR-449a |
| miR-548ai, miR-548ak, miR-548am, miR-548ap-5p, |  | miR-449b |
| miR-548aq-5p, miR-548ar-5p, miR-548as-5p, miR-548au-5p, |  | miR-454 |
| miR-5583-3p, miR-5584-5p, miR-559, miR-5692b, |  | miR-590-3p |
| miR-5692c, miR-5693, miR-5694, miR-570-5p, |  | miR-96 |
| miR-5700, miR-590-3p, miR-599, miR-93-5p, |  |  |

# Supplementary Data

List of 16 candidate miRNAs were selected by the selection criteria.

| miR-454 | miR-130a | miR-130b | miR-27a |
| --- | --- | --- | --- |
| miR-27b | miR-301a | miR-301b | miR-34a |
| miR-182 | miR-24 | miR-449a | miR-34c-5p |
| miR-449b | miR-3666 | miR-4295 | miR-128 |

**3 Supplementary material**

**Table S1** Primer sequence for detecting mRNA expression.

|  | Target gene | Nucleotide Sequence | Product size (bp) |
| --- | --- | --- | --- |
| Forward | *RUNX2* | 5’-AAC CCA GAA GGC ACA GAC AG-3’ | 192 |
| Reverse | *RUNX2* | 5’-GCC TGG GGT CTG TAA TCT GA-3’ |  |
| Forward | *Osterix* | 5’-TGC TTG AGG AGG AAG TTC AC-3’ | 114 |
| Reverse | *Osterix* | 5’-CTG CTT TGC CCA GAG TTG TT-3’ |  |
| Forward | *C/EBPβ* | 5’-TTT GTC CAA ACC AAC CGC AC-3’ | 154 |
| Reverse | *C/EBPβ* | 5’-GCA TCA ACT TCG AAA CCG GC-3’ |  |
| Forward | *PPARγ* | 5’-TGA AGG ATG CAA GGG TTT CT-3’ | 200 |
| Reverse | *PPARγ* | 5’-CCA ACA GCT TCT CCT TCT CG-3’ |  |
| Forward | *GAPDH* | 5’-CAA CTA CAT GGT TTA CAT GTT CCA A-3’ | 206 |
| Reverse | *GAPDH* | 5’-CAG CCT TCT CCA TGG TGG T-3’ |  |

**Table S2** Primers for detecting miRNAs expression via real-time RT-PCR. Bold letters denote the seed match sequence that is specific to *PPARγ*.

| **Type** | **Assay name** | **Nucleotide Sequence** |
| --- | --- | --- |
| Target miRNA | hsa-miR-130a-3p | **CAGUGCAAU**GUUAAAAGGGCAU |
| Target miRNA | hsa-miR-27b-3p | **UUCACAGUGG**CUAAGUUCUGC |
| Endogenous control | U6 snRNA | GTGCTCGCTTCGGCAGCACATATACTAAAA  TTGGAACGATACAGAGAAGATTAGCATGGC  CCCTGCGCAAGGATGACACGCAAATTCGTG  AAGCGTTCCATATTTT |
